# EBV-Tonsillitis with superinfection involving *Staphylococcus aureus* and *Prevotella Oris* leading to life-threatening bleeding in a 13-year-old girl: a case report

**DOI:** 10.1186/s12887-025-06441-7

**Published:** 2025-12-22

**Authors:** Janina Soler Wenglein, Thomas Boesing, Dennis Nordhoff, Burkhard Feidicker, Lars-Uwe Scholtz, Eckard Hamelmann

**Affiliations:** 1 Department of Pediatrics, Protestant Hospital of the Bethel Foundation, University Hospital OWL, Bielefeld University, Bielefeld, Germany; 2 Medical Faculty OWL, Bielefeld University, Bielefeld, Germany; 3 Clinic for Vascular Medicine, Protestant Hospital of the Bethel Foundation, Bielefeld, Germany; 4 Department of Otorhinolaryngology, Head and Neck Surgery, University Hospital OWL, Bielefeld University, Bielefeld, Germany

**Keywords:** Case report, Prevotella oris, Intensive care, EBV, Necrosis, Maxillofacial infections, Airway obstruction, PICU

## Abstract

**Background:**

Viral infections of the upper airways are common in children and adolescents, often presenting with mild symptoms and typically resolving without the need for hospitalization. Epstein-Barr virus (EBV), a gammaherpesvirus, can cause diverse clinical syndromes and transient immune dysregulation, potentially predisposing to bacterial superinfection.

**Case presentation:**

We report a 13-year-old girl with critical upper airway obstruction due to necrotizing ulcerative oropharyngitis in the context of an EBV infection, with superinfection by *Prevotella oris* and *methicillin-sensitive Staphylococcus aureus* (MSSA) isolated from operative tissue specimens. Within a very short time, the patient developed EBV-associated nephritis and pneumonia, severe sepsis, and acute respiratory distress syndrome (ARDS). Extensive soft-tissue necrosis precipitated life-threatening hemorrhage from the right external carotid artery followed by a second hemorrhage on the left, requiring coil embolization and ligation. Additionally, the patient experienced neurological complications due to thiamine deficiency, contributing to a complex and prolonged recovery process. Despite the severity of her illness, multidisciplinary management enabled stabilization and eventually discharge to a rehabilitation facility.

**Conclusion:**

To our knowledge, fulminant necrotizing oropharyngitis with bilateral external carotid hemorrhage during acute EBV infection in an immunocompetent adolescent is exceedingly rare. This highlights the importance of considering anaerobic oral colonizers as potential pathogens in maxillofacial infections. Clinicians should reassess for bacterial superinfection when EBV courses are protracted or deteriorating.

**Supplementary Information:**

The online version contains supplementary material available at 10.1186/s12887-025-06441-7.

## Background

Viral infections of the upper airways are common and typically mild in childhood and adolescence. Usually, the course of disease does not require hospitalization. However, severe cases can occur, especially in children with pre-existing conditions. Numerous viral pathogens can cause clinical symptoms in children and adolescents, including adenovirus, rhinovirus, enterovirus, human papillomavirus, parechovirus, Epstein-Barr virus (EBV), and others. These infections often go unnoticed, with only seroconversion indicating that an immune response has taken place. The seroprevalence of EBV is about 96% in adults [1].

EBV is a human gammaherpesvirus, known to cause a broad spectrum of disease in immunocompetent and immunocompromised patients, including nephritis, pneumonia, chronic fatigue, and Burkitt lymphoma. Its immunomodulatory effects include evasion of cellular antiviral mechanisms [2]. Additionally, EBV can switch between active and latent phases, potentially leading to long-term courses of chronically relapsing EBV infections with association to systemic autoimmune diseases [3]. Viral illnesses can facilitate secondary bacterial infection, enabling ordinarily commensal or environmental microbes to become pathogenic [4]. EBV favors bacterial superinfections by various mechanisms: interactions with oral bacteria [5], transient neutropenia [6], impairment of granulocyte function [7, 8] and T-cell alterations [9] may aggravate or promote susceptibility to bacterial diseases.

*Prevotella oris* belongs to the *Prevotella* genus: gram-negative obligate anaerobic bacilli that commonly act as commensals of the oral cavity and gastrointestinal tract and seldom lead to disease [10]. However, in the setting of immune perturbation or concomitant acute infection, they can lead to severe clinical pictures. In particular, (odontogenic) abscesses [11–15], pneumonia [16], pleural empyema [10, 17], as well as pericarditis [18], and bacteremia [19] have been reported.

Following the CARE guidelines [20], we present the case of a 13-year-old girl with severe necrotizing ulcerative oropharyngitis resulting from acute EBV infection with operative tissue cultures yielding *Prevotella oris* and *methicillin-sensitive Staphylococcus aureus* (MSSA), requiring prolonged intensive care and mechanical ventilation from day one due to upper airway obstruction. The course of disease included life-threatening hemorrhage from the right external carotid artery, as well as further complications with need of mechanical ventilation, loss of ability to swallow, and cosmetic disfigurement.

### Ethics and patients’ consent

Ethics approval was not required for this case report. The patient and her parents consented to the publication of this case report, and all provided details and illustrations.

## Case presentation

The patient was admitted to our hospital, the children’s clinic of the Protestant Hospital of the Bethel Foundation, University Hospital OWL, as an emergency by ambulance in March 2023 and was discharged in May 2023 to neurorehabilitation. At admission, her parents stated that she had been ill for about three weeks. Initially, the patient suffered from flu-like symptoms with a cough and fever for a week. Following a brief period of improvement, she showed increasing difficulties with swallowing, a sore throat, halitosis (fetor oris), and fever for the subsequent two weeks. Prior to admission, acute EBV infection was confirmed in the outpatient setting by serology. The night before hospital admission, she began spitting up blood and experienced severe breathing difficulties. Up to this point, no underlying diseases or chronic conditions were known. There was also no known relevant family history.

On first examination, the patient was conscious and showed cervical lymphadenopathy, and critical respiratory distress with inspiratory stridor and retractions. She was co-operative but aphonic and had prominent halitosis. Vital signs included a temperature of 38.3° C, a heart rate of 153/minute, oxygenation of 96% under 12 L/min oxygen supplementation via respiratory bag-mask, body weight 53.8 kg and blood pressure of 98/64 mmHg. The evident acute life-threatening obstruction of the upper airways and compromised circulation led to a clinical diagnosis of sepsis, and our patient was immediately admitted to the pediatric intensive care unit (PICU). Given impending airway failure, the airway was secured via endoscopic intubation, and sepsis management was initiated without delay. In the course of the septic illness, the patient showed vasopressor-dependent circulatory shock during inpatient life support measures. We therefore addressed circulatory support with volume resuscitation and vasopressor support. This status subsequently persisted with varying need for circulatory support over the following days. The clinical course is presented in Table 1(timeline of main symptoms and clinical course during intensive care treatment).

**Table 1:** Timeline of main symptoms and clinical course during intensive care treatment

Week	Main symptoms	Management and Findings
Week 1	Hospital admission with acute shortness of breath, severe airway obstruction, cervical lymphadenopathy, fever, spitting blood, circulatory insufficiency	Emergency admission, endoscopic airway management, initiation of sepsis treatment with antibiotics, volume resuscitation, and catecholamine therapy; diagnostic work-up including blood cultures, oral and throat swabs, analysis of tracheal secretions for bacterial, fungal, and viral pathogens, blood samples for viral PCR, and tissue biopsies
	Local necrosis of the upper airways	Repeated interventional irrigation and ablation
Week 2	Continuing catecholamine-dependent circulatory insufficiency, necrotizing and swollen tissues, inability to swallow	Supportive care for circulatory insufficiency with fluids and catecholamines; *Staphylococcus aureus* detected
	Day 9: Life-threatening bleeding from the right external carotid artery and further advanced necrotizing ulcerative oropharyngitis	Emergency surgical ligation of right external carotid artery, debridement of necrotic tissue, mass blood transfusion
Week 3	Continued need for invasive ventilation	Surgical installation of a tracheostomy
	Lack of oral nourishability	Gastrostomy
	Second vascular haemorrhage (left maxillary artery)	Coiling of the maxillary artery and vascular ligation of the left external carotid artery
	Further progression of soft tissue necrosis	Complete loss of pharyngeal lymphatic tissue, soft palate, and epiglottis with some tissue recovery and improved blood supply
Week 4	Stabilization of the circulatory system	Final cessation of catecholamine therapy on day 22 of hospitalization
Week 5	Delirium, post intensive care critical illness myopathy, generalized deceleration, low-normal thiamine level	Suspected Wernicke’s encephalopathy and initiation of high-dose thiamine therapy
Week 6	Decreasing need for mechanical ventilation	Continuous spontaneous breathing via tracheal cannula from day 42
	Persistent confusion and disorientation	Medication with haloperidol and clonidine; Environmental measures to stabilize routine, and close nursing support
Week 8	Pain in the lower extremities, especially feet, and only intermittent continence, with increasingly improved vigilance	Treatment of neuropathic pain with pregabalin; continuation of intensive physiotherapy
Week 10	Persistent neuromotor-psychological stress pattern, reduced strength in lower extremities, hypersensitivity of soles, neuropathic pain, terminal nystagmus, intermittent continence	Transfer for further complex neurorehabilitative treatment

Because of the typical clinical findings of upper airway obstruction with swollen tissues and dysfunction of laryngeal structures, as well as lymphadenopathy, we suspected an acute EBV infection with bacterial septic superinfection and immediately started a broad antibiotic therapy, with cefuroxime 90 mg/kg body weight in three doses per day, clindamycin 40 mg per kg body weight in three doses per day and metronidazole 30 mg/kg body weight in three doses per day. A comprehensive diagnostic work-up was performed, including blood cultures, oral and throat swabs, and analyses of tracheal secretions for bacterial, fungal, and viral pathogens. Blood samples for viral PCR and tissue biopsies were also obtained. Operative tissue cultures from necrotic oropharyngeal mucosa/abscess cavities yielded *MSSA* (spa type t084, clonal lineage ST15) and *Prevotella oris.* Since the detection was based on direct culture from surgical material, we assumed these organisms to be pathogenic. Both pathogens mentioned were further detected in throat swabs and tracheal secretions. A classification of the *Prevotella oris* strain was not possible. Organisms recovered in low burden from superficial swabs or tracheal secretions (*Staphylococcus epidermidis*,* Streptococcus anginosus*,* Pseudomonas aeruginosa*,* Finegoldia magna*,* Escherichia coli*) were interpreted as colonizers/overgrowth. We further detected picornavirus and *Candida albicans* in respiratory samples. Given disease severity, liposomal amphotericin B was administered at 5 mg/kg once daily initially and continued therapeutically after Candida confirmation.

**Fig. 1:**
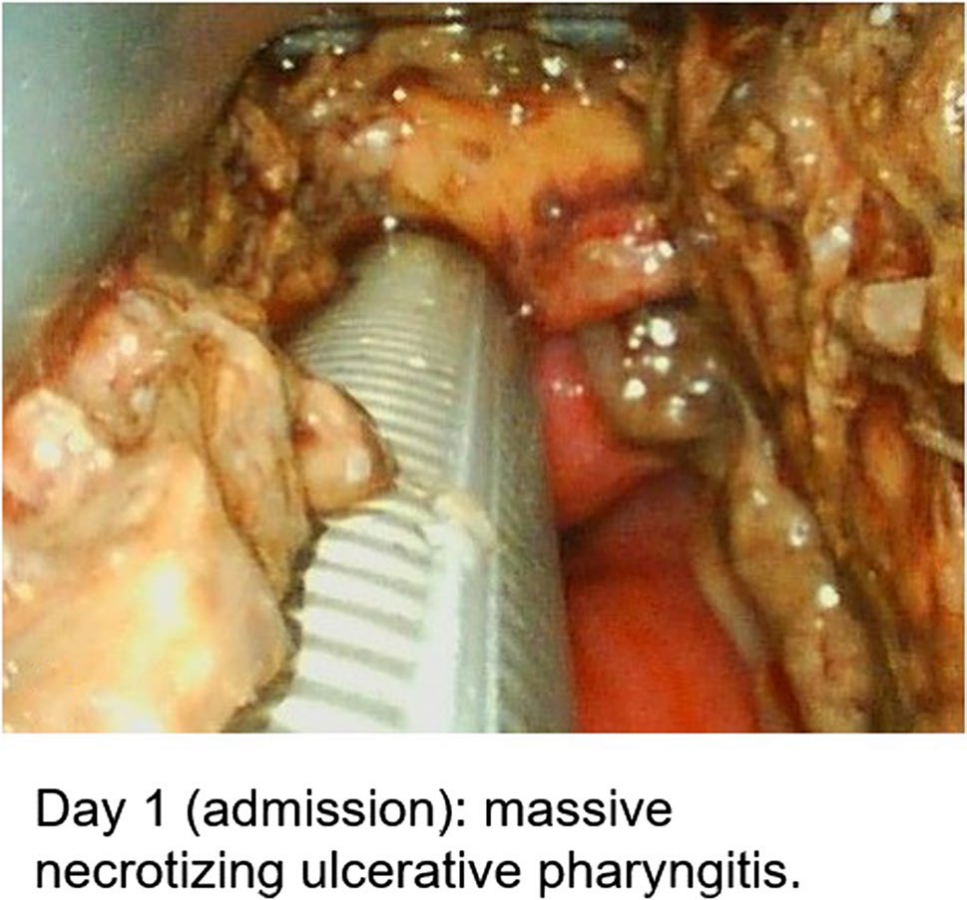
Laryngoscopic view on the course of disease from day 1 to day 49

**Fig. 2:**
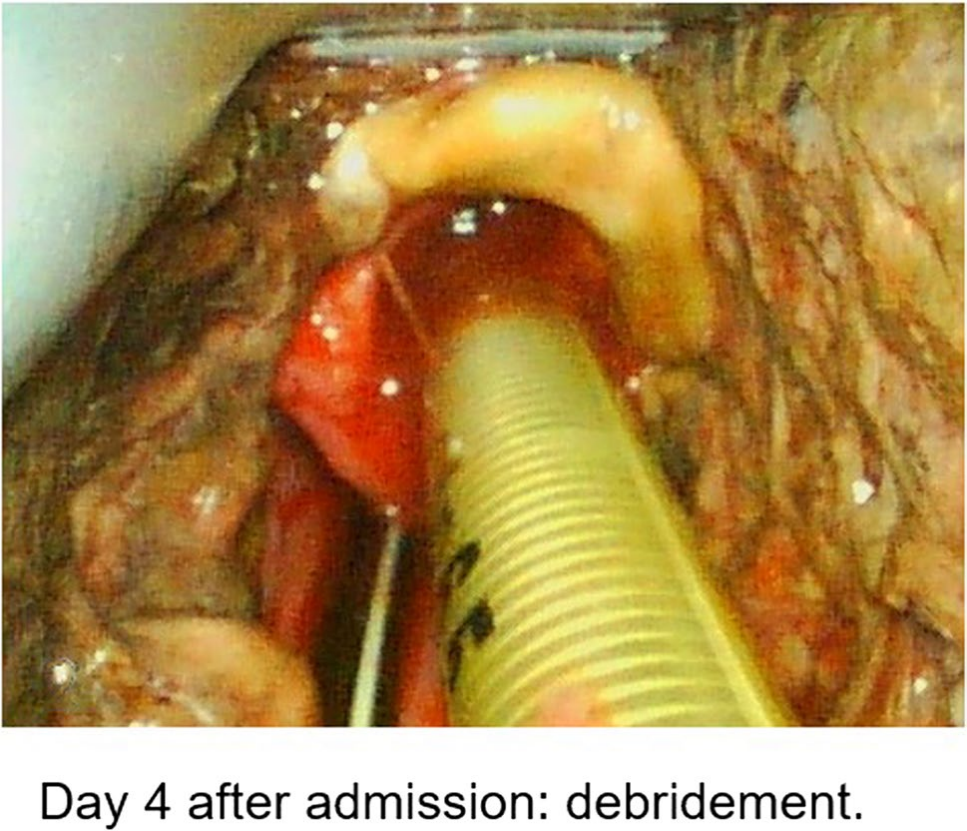
Laryngoscopic view on the course of disease from day 1 to day 49

**Fig. 3:**
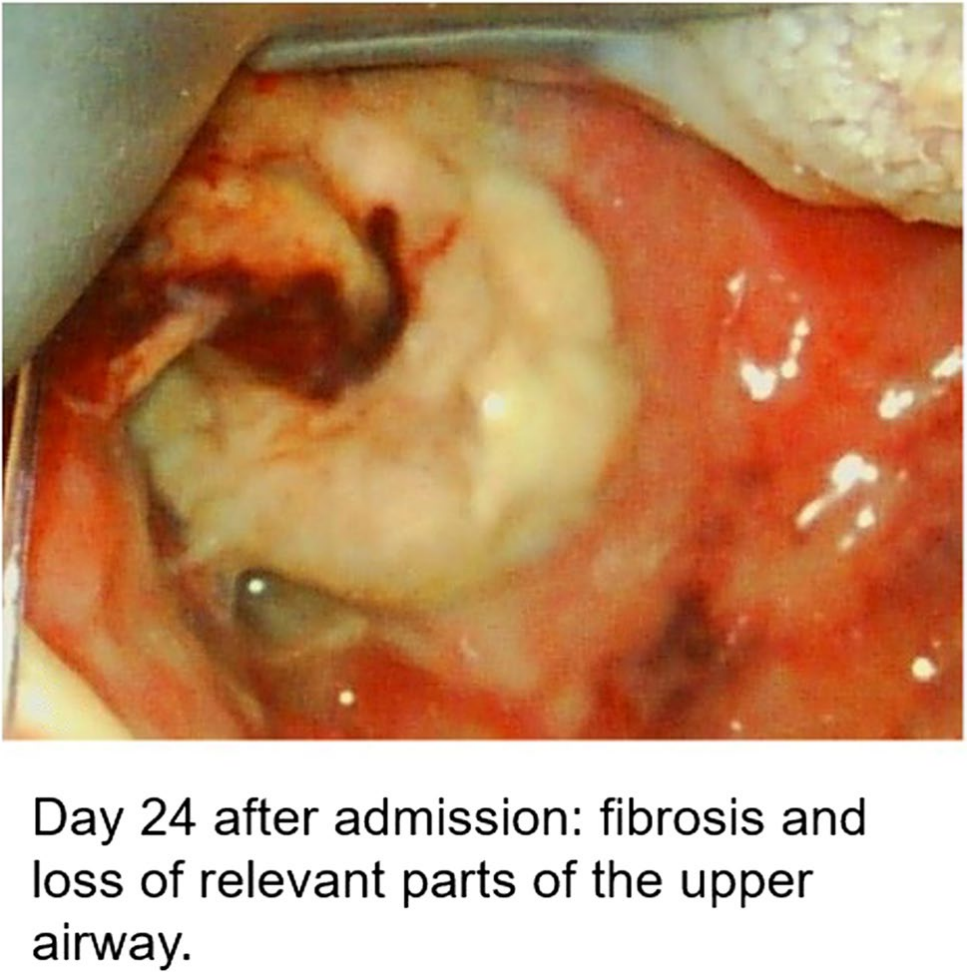
Laryngoscopic view on the course of disease from day 1 to day 49

**Fig. 4:**
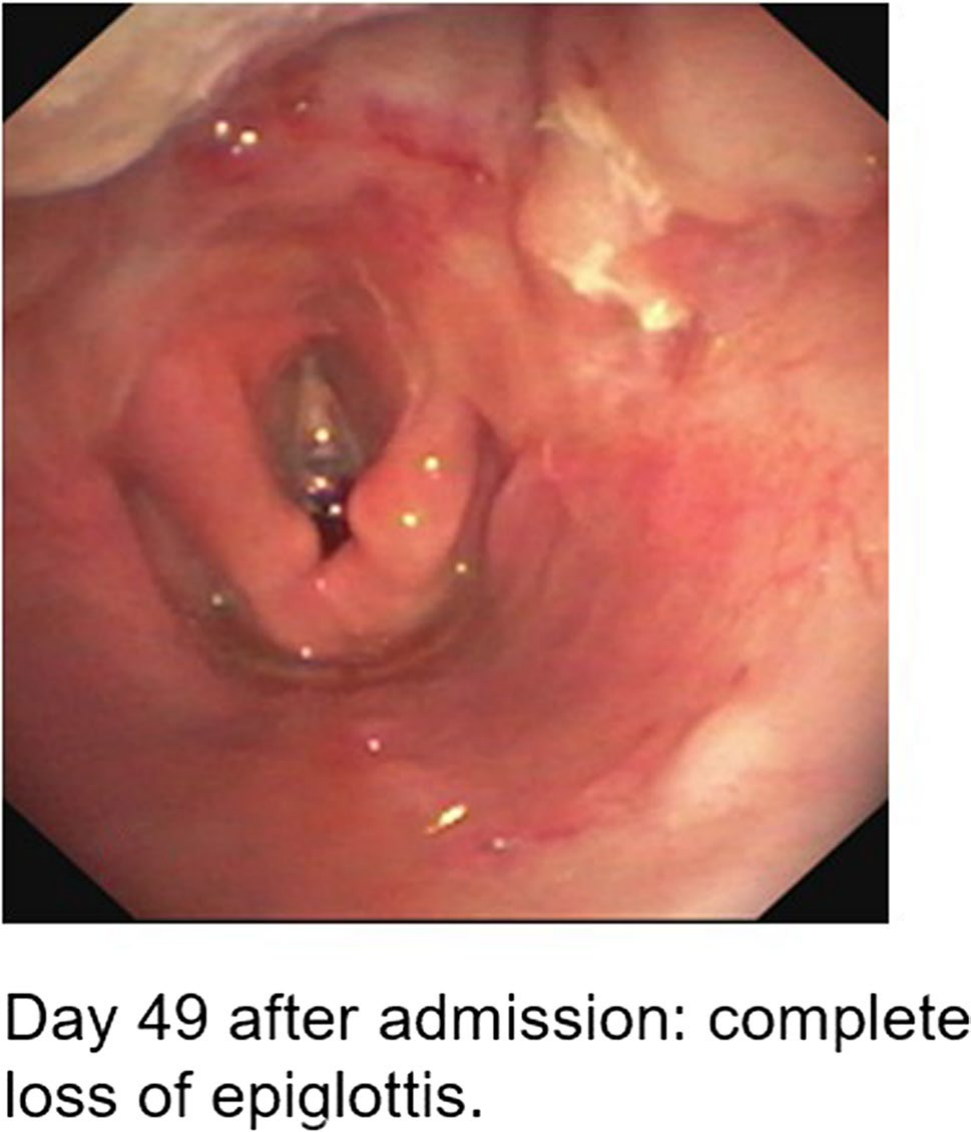
Laryngoscopic view on the course of disease from day 1 to day 49

**Fig. 5:**
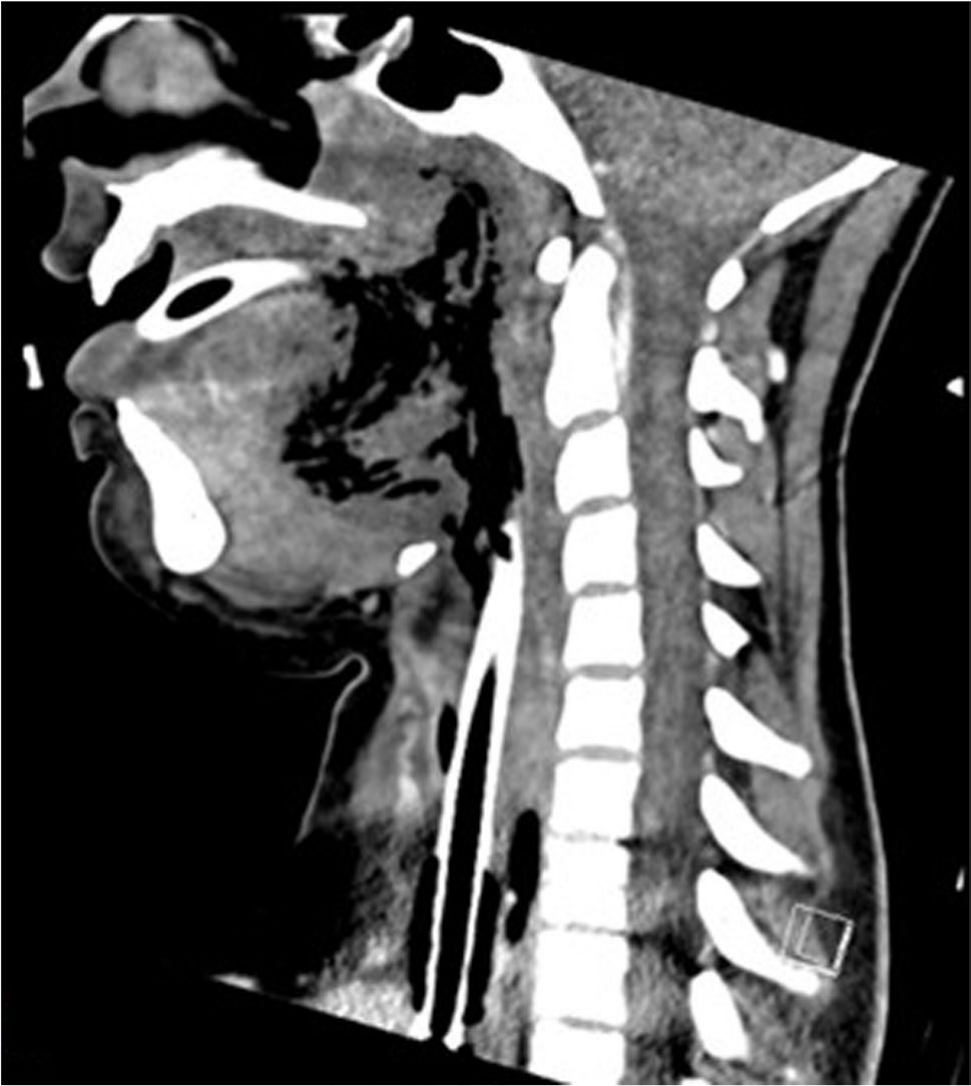
Contrast-enhanced CT scan revealing deep neck soft-tissue inflammation with necrosis extending toward the carotid spaces

**Fig. 6:**
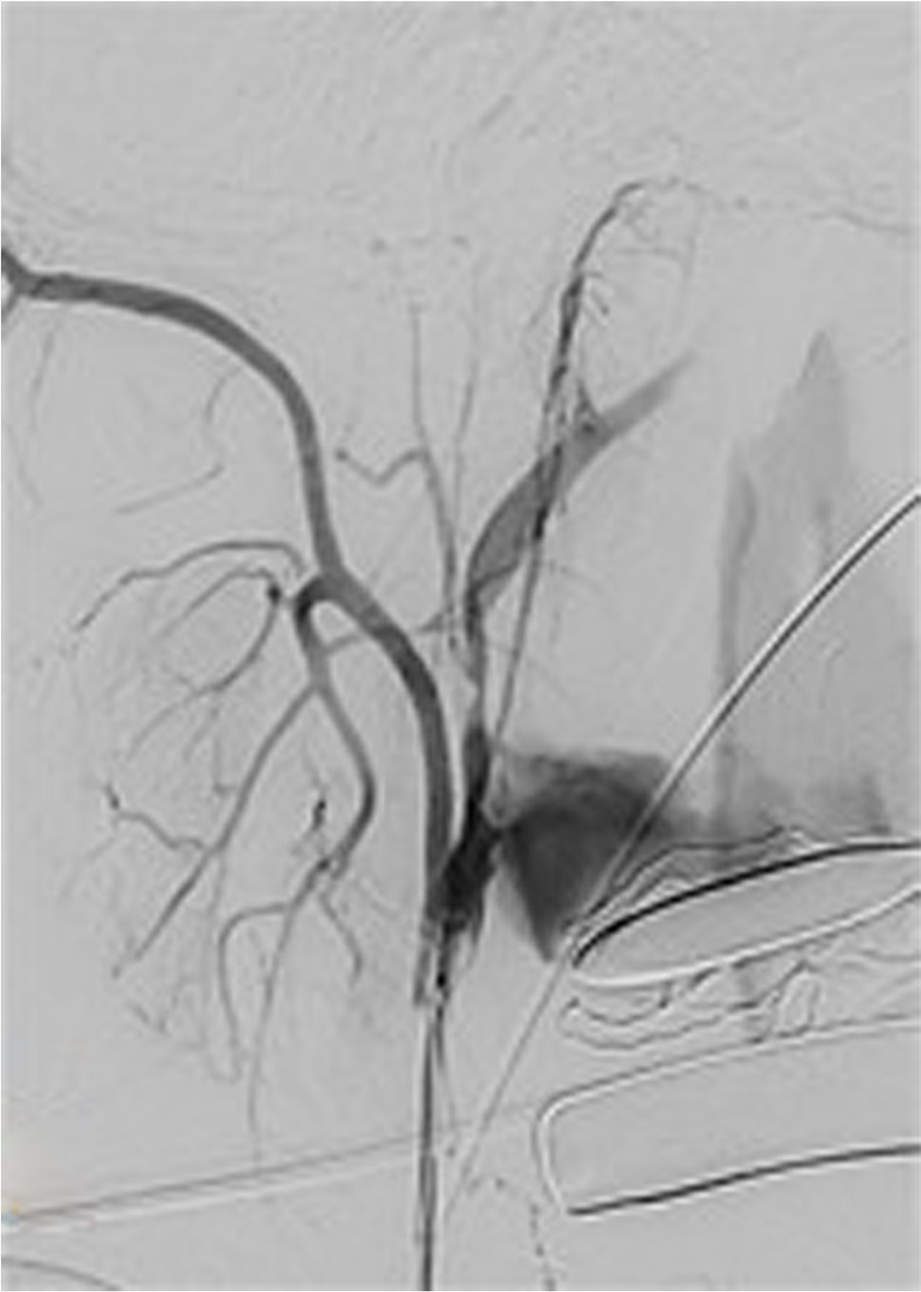
Coil embolization of the left external carotid artery

**Fig. 7:**
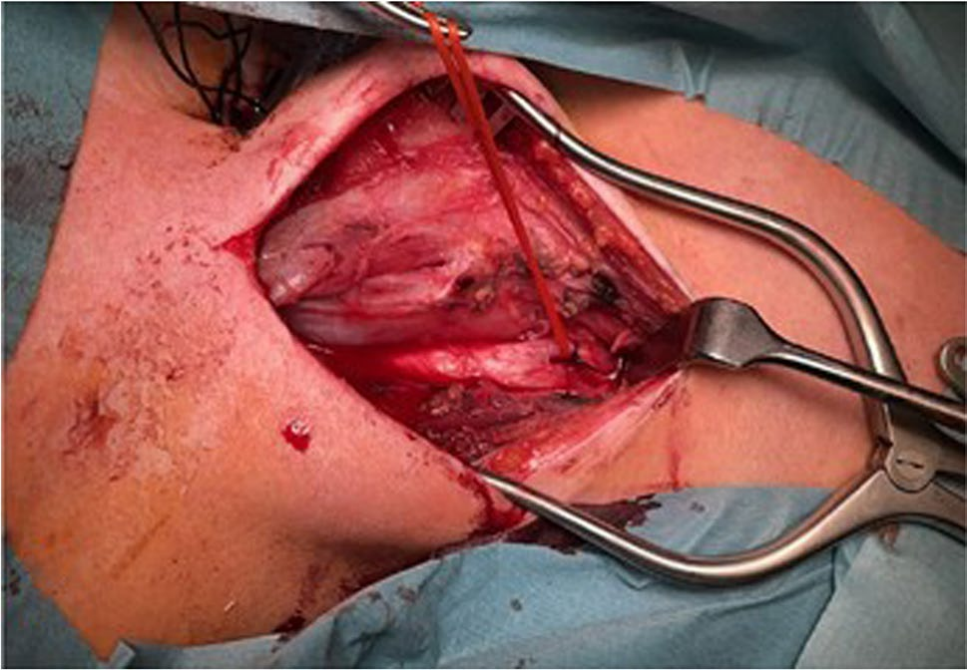
Ligation of the left external carotid artery

During intubation, we clinically diagnosed extensive necrotizing-ulcerative oropharyngitis [see Figs. 1, 2, 3, and Fig. 4, course of disease]. A contrast-enhanced CT scan [Figure 5] revealed deep neck soft-tissue inflammation with necrosis extending toward the carotid spaces. In collaboration with pediatric, vascular, and ENT surgeons, repeated debridement of the necrotized mucosal areas was initially performed via flexible endoscopy and later through direct laryngoscopic view [see Fig. 1, 2, 3 and Fig. 4]. Because the patient was hemodynamically unstable, no further open surgical management was feasible initially. In close consultation with the involved surgical specialties, we performed temporizing endoscopic debridement and planned staged external exploration following initial stabilization. But one week after admission, catastrophic hemorrhage occurred from the right external carotid artery, necessitating emergent ligation and further external surgical debridement. In the course of this bleeding, circulatory instability was excessive, and massive transfusion of blood products was necessary. During a later performed tracheostomy, a second vascular hemorrhage occurred, this time from the left maxillary and occipital arterial branches with more proximal involvement. The left external carotid artery was first coil embolized and then ligated [see Figs. 6 and 7]. Residual upper airway soft tissues were severely reduced: The patient lost more than two-thirds of the mobile tongue, tonsillar tissue, large areas of oral mucosa, and the entire epiglottis [Figures 1, 2, 3 and Fig. 4].

Due to the extensive findings, oral feeding was deemed impossible, leading to a surgical gastrostomy performed alongside the tracheostomy. Despite this, food intake and transport remained impaired. With gradual reduction of opioid sedation (sufentanil) and initiation of prokinetic therapy with erythromycin, enteral feeds were advanced; parenteral nutrition was used in the interim. Initially, the patient was kept nil per os with intravenous crystalloids. On day 3 of PICU treatment, small‑volume oral rehydration (≤ 200 mL/day) was introduced. Because advancement of enteral nutrition was not feasible, parenteral nutrition was started using an amino acid–glucose solution without lipids at 480 mL/day to stay within the overall fluid allowance required for ongoing PICU therapies. This provided 683 kJ/day and approximately one quarter of the calculated amino acid target (full daily requirement ≈ 2150 mL of the standard solution). Glucose and electrolytes were delivered via additional infusions. As tolerance improved, the parenteral rate was increased and switched to a higher–energy‑density preparation, with brief interruptions around surgical procedures. From week 2, total energy delivery was nearly doubled. Throughout, the gut was primed with small‑volume enteral electrolyte solution via feeding tube. By one month after PICU admission, a specialized enteral formula was established. Micronutrients, including vitamins and trace elements, were incorporated into the parenteral regimen as volume permitted and as the formulation was escalated. Overall, meeting full nutritional requirements throughout the first month was not feasible given the clinical constraints. A thiamine deficiency, linked to sepsis and the challenging nutritional situation, led to clinical signs of Wernicke encephalopathy, compounded by critical illness polyneuropathy and PICU delirium after prolonged sedation. The EEG showed generalized slowing of brain activity. A cranial magnetic resonance tomography (cMRI) and low-normal vitamin B1 levels confirmed the diagnosis. We note that serum thiamine can be an insensitive marker and diagnosis relied on clinical-radiologic correlation and response to therapy. Following high-dose thiamine therapy and appropriate delirium management, the neurological symptoms improved, and a follow-up MRI four weeks later showed nearly complete resolution of the changes.

### Patient perspective

The patient reported a positive relationship with the healthcare team, although she experienced severe depression due to the loss of functional autonomy in her daily life. Both the patient and her parents appreciated the transparent communication. The parents expressed feeling emotionally supported by the intensive care team, especially during the most critical phases of their daughter’s illness. The family experienced the medical care during rehabilitation as less helpful, partly because the need for parental presence while both parents worked was almost impossible to meet. Eventually, rehabilitation was canceled prematurely. In the long term, the patient became psychologically destabilized with increasing recognition of her everyday limitations. The patient’s neuropathy and depressive symptoms made it considerably more difficult for her to practice her gait. Oral hygiene was also systematically neglected. Repeated inpatient stays at our clinic were therefore necessary, including complex psychosomatic treatment for four months in our hospital in 2024.

The psychological long-term difficulties of our patient underline the importance of early recognition of psychosocial support. In our intensive care unit, we strive to take this need into account by integrating social workers, psychologists, music and occupational therapists into our intensive care therapy at an early stage and offer regular dialogue sessions for relatives.

At present, the patient is increasingly independent in mobility and communicates with a speaking valve and computer-assisted tools.

## Discussion

This case involved a previously healthy 13-year-old girl presenting with severe upper airway compromise and septic symptoms. Based on the clinical presentation, several differential diagnoses were considered. Given the outpatient suspicion of acute EBV infection and her septic presentation, we initially presumed an EBV‑associated bacterial superinfection; Plaut–Vincent angina and Lemierre’s syndrome were also considered. The patient showed no evidence of purulent thrombophlebitis and no signs of septic emboli, as would have been expected in Lemierre’s syndrome. We also ruled out jugular vein thrombosis. Specifically, duplex ultrasonography and contrast-enhanced CT of the neck demonstrated patent internal jugular veins without mural thrombus or perivascular inflammatory changes. Aerobic and anaerobic cultures from operative tissue/abscess material yielded *Staphylococcus aureus* and *Prevotella oris*; no *Fusobacterium spp*. or spirochetes were detected by culture or targeted assays. The clinical course was dominated by rapidly progressive necrotizing infection and hemorrhage rather than the biphasic evolution with metastatic septic complications typical of Lemierre’s syndrome. Plaut–Vincent angina is usually a localized necrotizing ulcerative tonsillitis or gingivitis associated with fusiform bacilli and spirochetes and is characterized by limited systemic toxicity; in contrast, our patient had bilateral deep neck involvement with airway compromise and catastrophic hemorrhage, cultures were negative for spirochetes and *Fusobacterium*, and deep operative specimens grew *Prevotella oris* and MSSA. Taken together, these findings support a diagnosis of severe necrotizing cervicofacial infection in the setting of acute EBV-associated immune dysregulation, rather than Lemierre’s syndrome or Plaut–Vincent angina.

*Prevotella* species are gram-negative, obligate anaerobic bacilli and are recognized causes of oral and mediastinal infections [19], although *Prevotella oris* rarely leads to clinical symptoms. It is mainly reported in studies focusing on dental diseases or gut microbiota. Reports of severe infections are rare, but some cases of children with severe co-infections of *Prevotella* species and bacteria have been reported. They focus on buccal abscess [21], deep neck infection, Lemierre´s syndrome [22], and pulmonary abscess [15]. Coaggregation of bacteria plays a key role in oral infections [23]. *Staphylococcus aureus*, a common member of the oral cavity microbiota [24], is also a frequent cause of skin and soft tissue infections [25]. The isolation of MSSA and *Prevotella oris* from deep operative abscess tissue supports their role as co-pathogens. The severity of disease likely reflects synergism, with *S. aureus* contributing toxin-mediated tissue injury and *Prevotella oris* driving anaerobic necrotizing infection typical of necrotizing gingival disease. We acknowledge that quantitative cultures, virulence assays, and genomic sequencing of *Prevotella oris* were not performed, which limits definitive causal attribution.

The multisystemic effects of the reported severe septic illness with the development of an PICU delirium, critical illness polyneuropathy, and myopathy after prolonged intensive care were further compounded by a challenging nutritional situation, leading to a Wernicke encephalopathy due to a relative lack of vitamin B1. Thiamine deficiency can be prevalent in critically ill patients, particularly those with sepsis [26], where it can contribute to lactic acidosis and organ injury [27, 28]. Data from an animal sepsis model suggest thiamine deficiency also contributes to TNF-alpha elevation and oxidative stress [29]. It is also common in children during intensive care treatment [30]. Despite low-normal serum thiamine, the clinical and radiological picture supported the diagnosis, and neurological improvement followed high-dose thiamine. We recommend routine nutrition screening at least every 7–10 days in prolonged pediatric sepsis and early consideration of empiric thiamine supplementation in high-risk cases. After 10 weeks of hospital treatment, the patient was discharged to neurorehabilitation.

Limitations of our case report include a lack of organism quantification and sequencing for *Prevotella oris*, incomplete documentation of serial nutrition metrics, and EBV serology results not being available at initial submission.

## Conclusion

This case demonstrates the rare but serious potential for EBV-related infections to escalate into severe, life-threatening conditions through bacterial superinfection. In our patient, the combination of *Staphylococcus aureus* and *Prevotella oris* isolated from deep operative tissue led to necrotizing ulcerative oropharyngitis, severe sepsis, and catastrophic hemorrhages from the external carotid arteries, all of which required intensive surgical and medical interventions. These severe complications emphasize the need to consider the potential temporary immune dysfunction under EBV infection and the risk of bacterial superinfection in a protracted course with clinical vigilance for anaerobic pathogens in deep neck infections and systematic nutrition/micronutrient monitoring during prolonged critical illness. To our knowledge, such fulminant necrotizing oropharyngitis with bilateral external carotid hemorrhage in an immunocompetent adolescent is exceedingly rare. The prolonged critical illness and nutritional deficiencies underscore the importance of regularly monitoring nutritional status in septic patients; based on the available literature we suggest at least every 10 days. Although the long-term quality of life for the patient is severely impacted, timely and comprehensive treatment allowed for stabilization and the opportunity for rehabilitation.

## Supplementary Information

Supplementary Material 1.

## Data Availability

All relevant data is included in the case report. Further information are available from the corresponding author on reasonable request.

## Abbreviations

ARDS: Acute respiratory distress syndrome
cMRI: Cranial magnetic resonance tomography
CT: Computed tomography
EBV: Epstein-Barr virus
EEG: Electroencephalography
MSSA: Methicillin-sensitive Staphylococcus aureus
PICU: Pediatric intensive care unit
