# Supplementary material for: EBV-Tonsillitis with superinfection involving Staphylococcus aureus and Prevotella Oris leading to life-threatening bleeding in a 13-year-old girl: a case report
Source: BMC Pediatr. 2025 Dec 22;26:64. doi: 10.1186/s12887-025-06441-7 (PMC12849362; doi:10.1186/s12887-025-06441-7)
Supplement: Supplementary file 1 — Supplementary Material 1. [file 12887_2025_6441_MOESM1_ESM.docx]

| Table 1 Timeline of main symptoms and clinical course during intensive care treatment | | |
| --- | --- | --- |
| Week | Main symptoms | Management and Findings |
| Week 1 | Hospital admission with acute shortness of breath, severe airway obstruction, cervical lymphadenopathy, fever, spitting blood, circulatory insufficiency | Emergency admission, endoscopic airway management, initiation of sepsis treatment with antibiotics, volume resuscitation, and catecholamine therapy; diagnostic work-up including blood cultures, oral and throat swabs, analysis of tracheal secretions for bacterial, fungal, and viral pathogens, blood samples for viral PCR, and tissue biopsies |
|  | Local necrosis of the upper airways | Repeated interventional irrigation and ablation |
| Week 2 | Continuing catecholamine-dependent circulatory insufficiency, necrotizing and swollen tissues, inability to swallow | Supportive care for circulatory insufficiency with fluids and catecholamines; *Staphylococcus aureus* detected; |
|  | Day 9: Life-threatening bleeding from the right external carotid artery and further advanced necrotizing ulcerative oropharyngitis | Emergency surgical ligation of right external carotid artery, debridement of necrotic tissue, mass blood transfusion |
| Week 3 | Continued need for invasive ventilation | Surgical installation of a tracheostomy |
|  | Lack of oral nourishability | Gastrostomy |
|  | Second vascular haemorrhage (left maxillary artery) | Coiling of the maxillary artery and vascular ligation of the left external carotid artery |
|  | Further progression of soft tissue necrosis | Complete loss of pharyngeal lymphatic tissue, soft palate, and epiglottis with some tissue recovery and improved blood supply |
| Week 4 | Stabilization of the circulatory system | Final cessation of catecholamine therapy on day 22 of hospitalization |
| Week 5 | Delirium, post intensive care critical illness myopathy, generalized deceleration, low-normal thiamine level | Suspected Wernicke's encephalopathy and initiation of high-dose thiamine therapy |
| Week 6 | Decreasing need for mechanical ventilation | Continuous spontaneous breathing via tracheal cannula from day 42 |
|  | Persistent confusion and disorientation | Medication with haloperidol and clonidine; Environmental measures to stabilize routine, and close nursing support |
| Week 8 | Pain in the lower extremities, especially feet, and only intermittent continence, with increasingly improved vigilance | Treatment of neuropathic pain with pregabalin; continuation of intensive physiotherapy |
| Week 10 | Persistent neuromotor-psychological stress pattern, reduced strength in lower extremities, hypersensitivity of soles, neuropathic pain, terminal nystagmus, intermittent continence | Transfer for further complex neurorehabilitative treatment |
